# Root morphology and physiological of their relationship with nitrogen uptake in wheat (*Triticum aestivum* L.)

**DOI:** 10.1016/j.heliyon.2024.e29283

**Published:** 2024-04-06

**Authors:** Bo Qu, Fujie Feng, Jun Di, Hafeez Noor

**Affiliations:** a Department of Biological Science and Technology, Jinzhong University, Shanxi, 030619, China; b College of Agriculture, Shanxi Agriculture University, Taigu, 030801, Shanxi, China

**Keywords:** Gene expressions, Nitrogen fertilizer, Photosynthetic characteristics, Root characteristics, Wheat

## Abstract

Nitrogen (N) application is believed to improve photosynthesis in flag leaf ultimately increase final yield. The main results at 20–30 days after anthesis, the activities of superoxide dismutase (SOD) and peroxidase (POD) and soluble protein in flag leaves of N150 were found to be the most effective. Increased root weight density, root length density and root volume density at flowering stage, up to 10.6 %, 15.0 %, respectively. The root weight density, root length density and root bulk density at flowering and mature stages were the highest at the N180. Delaying the senescence physiology of post flowering leaves in the middle, and late stage, photosynthesis of leaves in the middle and late stage, improving the light energy interception of wheat, and then improving the light energy utilization efficiency. The stomatal conductance of flag leaves 15–30 days after anthesis, the maximum potential photochemical efficiency 20–30 days after anthesis, and the photochemical quenching of flag leaves 25–30 days after anthesis, and improved the light energy utilization efficiency by 9.6%–11.1 %. Yunhan-20410 the gene expressions of *TaTZF1*, *TaNCY1*, *TaNCY3* and *TaAKaGall* in wheat flag leaves were significantly up-regulated YH-20410 gene expressions of N application treatment were significantly up-regulated compared with no N application treatment. The goal of high yield high efficiency, and high quality can be achieved by YH-20410 and combined to N180 kg ha^−1^. The senescence physiology and gene expression of post flowering leaves in the middle and late stage, prolonging the photosynthesis of leaves in the middle and late stage, improving the light energy interception of canopy, and then improving the light energy utilization efficiency.

## 1 Intodution

Wheat (*Triticum aestivum* L.) is the most widely used food crop in the world, with more than 40 % of the world's population relying on wheat as the main diet. In 2016, the global wheat planting area was about 216.03 million hectares, accounting for 30.7 % of the global grain planting area and ranking first in the global grain planting area [1]. The main wheat producing regions are located in Asia, which accounts for 45.6 % of the global wheat acreage. China wheat output accounts for 17.6 % of the world total wheat output, making it the largest wheat producer in the world [2]. The increase of extreme weather around the world, the yield of major wheat producing countries has been damaged, which directly affects the global wheat food security. On the whole, global wheat supply and demand have been in a tight balance for a long time [1]. Wheat yield is mainly affected by crop varieties and cultivation practices. N rate is one of the main cultivation measures in wheat production. The cultivation had great influence on the emergence rate, growth and development process, population quality, and yield of wheat [3]. Nitrogen fertilizer plays a crucial role in the formation of wheat yield, and increasing nitrogen fertilizer application is one of the main ways to increase wheat yield [4]. Although application of nitrogen fertilizer is an important measure to improve wheat yield, excessive and irrational application of nitrogen fertilizer will reduce N use efficiency and cause a series of problems such as environmental pollution [5]. Therefore, optimization of nitrogen fertilizer plays an important role in high yield and high efficiency production of wheat root growth. Root system is an important part of soil-plant system. Wheat root system plays an important role in the growth and development of wheat, and the development of its root system will directly affect the aboveground growth of wheat [6,7]. Nevertheless increasing the N content, the Net photosynthetic rate (*P*n), stomatal conductance (*g*s), transpiration rate (*Tr*), and Ci intercellular CO_2_ concentration as well as increasing the substomatal CO_2_ concentration (*C*_i_) are considered as the value of wheat stomatal restriction in different plant populations. It was reported that N also improved yield and components in wheat [8]. However, the process of nitrogen transport from wheat organs to grains is extremely complex in physiology. Field-scale studies have no way to determine which photosynthetic nitrogen, and stored nitrogen transport starts first, or even synchronously, but the transport ratio of photosynthetic nitrogen, and stored nitrogen can be calculated [7]. The effects on the net photosynthetic rate (*P*_N_) vary in seed types and at different growth stages. Current scenario of climate change such as high temperature requires development of wheat with improved leaf gas-exchange parameters. Wheat *P*_N_ which is not affected by high intensity of light, temperature, and low humidity [7,8]. The competition among plant individuals is mainly the competition between plant roots for soil moisture. Therefore, influenced by long-term natural conditions, the root system of crops growing in semi-arid rain-fed agricultural areas is usually too large [9]. However, many previous studies on root size and drought resistance of crops have different views. The majority of researchers have shown that only developed roots in the underground part of plants can make plants drought resistant [10]. The nutrients that can be absorbed by crops mainly come from soil background nutrients and fertilizers used. The results of long-term experiment in Lausanne, UK showed that 8.1%–20.5 % of the nitrogen applied to the soil by crops of the last season can be continued to be absorbed and utilized by crops of the next season [8,11]. The photosynthetic rate, maximum fluorescence, stomatal conductance maximum photochemical quantum yield of PSII (F_v_/F_m_), variable fluorescence, photochemical quenching coefficient (qP) and potential activity of PSII (Fv/Fm) of flag leaves at flowering stage, and the relative content of chlorophyll could be increased by 17.7 % [12]. The grain yield of wheat could be increased through the increase of biomass. Differences in light, temperature and other natural environmental conditions during wheat growth, and development as well differences in soil nutrients and soil texture, have a certain impact on dry matter accumulation and transport of wheat [13,14]. Under high-yield cultivation conditions, lodging resistance characteristics of wheat of different varieties types are different, and dry matter accumulation and yield of wheat are also different [15,16]. The appropriate nitrogen application rate can build a reasonable population structure of wheat, which is further conducive to the coordinated development of the three factors of wheat spike number, grain number per spike and 1000-grain weight [17]. A large number of studies have shown that there is a close relationship between aboveground nitrogen accumulation and floret number per spike of wheat at the early spike differet stage [18]. N application of 180 kg ha^−1^ nitrogen fertilizer in shuidi wheat has better population quality, which is reflected in higher tiller rate, higher leaf area index at booting stage and filling stage, higher dry matter accumulation after anthesis, and higher spike number and yield of wheat [19].

The objectives of this study were (1) photosynthetic characteristics of ear leaf (2) root morphological characters, root activity, and gene expressions in root and (3) improving the light energy utilization efficiency and grain yield. This paper will offer a basis for further increasing nitrogen fertilizer application, and achieving a high yield of winter wheat in northeast China.

## 2 Materials and methods

The greenhouse experiment was conducted in the wheat experimental base of Shanxi agricultural university in Taigu, Shanxi Province, China (E112°34 'E, N37°25' N) from 2020 to 2022. The pond was planted in a pool 2 m deep, separated by concrete walls with a thickness of 20 cm, and an insulation layer of 10 cm added to the outer walls.

### 2.1 Experimental design

The experiment had a split-split-plot design with three replications. From 2020 to 2022. Subplots were treated with two varieties (Yunhan-20410’ and ‘Yunhan-618) were obtained from the Taigu Agriculture Bureau, Taigu, China. Sub-subplots were four nitrogen rate (N0, N180, N240, and N300 kg ha^−1^), respectively. The area of each plot was 2 m × 4 m = 8 m^2^, repeat 3 times. Before sowing Pure P_2_O_5_ and K_2_O were applied at the rate of 150, and 75 kg ha^−1^, respectively. Applied nitrogen fertilizer to the base fertilizer. The planted in manual with row spacing of 20 cm and sowing quantity of 225 kg ha^−1^. Irrigation was applied using a drip irrigation system, with application of 50 mm each time as measured with a water meter. The greenhouse was kept free from insects, pests, and diseases using pesticides as needed. During whole growing season, weed was well controlled by hand.

### 2.2 Photosynthetic pigment and chlorophyll fluorescence parameters

The chlorophyll fluorescence characteristics of wheat flag leaves were measured at the anthesis stage. Portable modulated chlorophyll fluorimeter (PAM-2500, Heinz Walz GmbH, Pfullingen, Germany) was used to determine the chlorophyll fluorescence induction kinetics parameters of fully expanded wheat flag leaves with consistent growth, and light exposure from 8:00am to 12:00am every day. The wheat was kept in the dark for at least 20 min. After dark treatment, Chl fluorescence induction curves were measured using plant efficiency analyzer (PEA) with red irradiance of 3000 μmol (photon) m^−2^ s^−1^. Measurements were performed at 12:00 and 17:00 h on the same day. The SPAD-502 measurements were conducted in the field between 10:00 and 16:00 h. The adaxial side of the leaves was always placed toward the emitting window of the instrument, and major veins were avoided. The area of the punched wheat leaf material was determined using an area meter (Delta-T Devices Ltd., Cambridge, UK), while two circular 1.0 cm diameter leaf discs from one side on the midrib were punched for wheat. The spatial distribution of the punched leaf material matched the distribution of the single-photon avalanche diode SPAD-502 [20].

### 2.3 Physiological and biochemical traits measurement

The contents of malondialdehyde (MDA), and osmotic solutes in fresh leaves were analyzed at the end of the drought stress period (at 5, 10, 15, 20, 25 and 30 days after anthesis). For preparing the crude extracts, 0.5 g of fresh leaves from three replicates in each treatment were homogenized in a prechilled mortar and pestle using 10 mL of 50 mmol L^−1^ phosphate buffer (pH 7.8) containing 1 % of soluble polyvinyl pyrrolidone (PVP). The ho-mogenates were then centrifuged at 10,000×*g* for 20 min at 4 °C, and the resulting super-natants were used for enzyme activity and biochemical assays. The content of MDA was determined using 2-thiobarbituric acid (TBA) [21]. Briefly, 2 mL extract solution was added to 2 mL of 0.5 % TBA (dissolved with 15 % trichloroacetic acid) and incubated in a water bath at 95 °C for 30 min. After centrifugation (10,000×*g* for 10 min), the MDA content was determined as mmol g^−1^ FW (fresh weight) by measuring the absorbance at wavelengths of 450 nm, 532 nm, and 600 nm using a spectrophotometer [21].

### 2.4 Sampling of roots and determination of root parameters

A root was used to sample the root system of wheat at flowering and maturity stages, with a sampling depth of 0.6 m. Three sampling points were randomly selected in the plot. After the root samples were taken, the roots were loaded into a 100-mesh nylon mesh with a written number, soaked in water for 1h, and then washed with tap water to remove impurities in the wheat root samples. Filter paper was used to drain water from the surface of the root system. The treated root system was divided into two parts. The second sample was passed through a root scanner (Epson V850 Pro; After scanning and imaging, Seiko Epson Corp, Japan) was used for analysis with root analysis software (WinRHIZO 2017, Regent Instruments, Canada). After that, this part of root was lowered and placed in a drying box. The dry weight of dried root samples was weighed and recorded. The other part was frozen as fresh root samples in a low temperature refrigerator (−20 °C to −25 °C) to determine the content of super oxide dismutase (SOD) [22], root triphenyl tetrazolium chloride (TTC) reduction intensity [2], and malondialdehyde (MDA) [23].

### 2.5 Root length decay rate

Root length decay rate = (root length in flowering stage - root length in mature stage)/root length in flowering stage × 100 %

### 2.6 Determination of yield and composition

At the mature stage of wheat, the edge rows of plots were removed, and the effective number of ears in 0.667 m^2^ representative wheat quadrat was investigated in each plot. Then 20 spikelets were randomly selected in each plot to investigate the number of grains per ear. Three 0.667 m^2^ quadrats of wheat from each plot were harvested and cut for yield and threshing. Grain moisture analyzer (PM8188A, Kett Electric Laboratory Tokyo, Japan) was used to determine the water content of wheat grains. The yield was calculated according to the standard water content (13 %), and the 1000-grain weight of wheat was investigated, and each treatment was repeated three times.

### 2.7 Reverse transcription

Aidlab reverse transcription kit was used (TUREscript 1st Stand *cDNA* SYNTHESIS Kit) (Table 1). Aidlab reverse transcription kit was used after holding at 42 °C for 40 min, heating at 65 °C for 10 min, cooling on the ice, centrifugation can be used. After the reaction, cDNA was obtained and stored at −80 °C.

**Table 1:** *PCR* reaction buffer.

Component	Volume
Total RNA	1 μg
5 × RT Reaction Mix	4 μL
Rondam primer/oligodT	0.5 μL
N6	0.5 μL
TUREscript H^−^ RTase/RI Mix	0.8 μL
RNase Free ddH_2_O	Up to 20 μl

### 2.8 Using fluorescence quantitative qPCR

Primer design primers, 5.0 in (Table 2) for the primers used in the experiment. The expression of target genes was analyzed by ABI 7500 real-time fluorescence quantitative PCR. A 20 μL PCR reaction system was prepared (Table 3). The *qPCR* system was as followsReaction procedure: 95 °C predenaturation 10s, 94 °C denaturation 5s, annealing 30s, a total of 39 cycles. After the reaction, the experimental results were analyzed by 2^−Δ^Ct algorithm.

**Table 2:** qPCR reaction buffer.

*qPCR*	Loading volume (μL)
*2×SYBR® Green Supermix*	5
*Forward primer*	0.5
*Reverse primer*	0.5
*cDNA*	1
*ddH*_*2*_*O*	3

**Table 3:** The primers of *qPCR*.

*Gene*	Primer sequence (5′→3′)	Accession No
*TaTZF1*	F:5′-TTCCGCATGTACGACTTC-3′ R:5′-GGAGTAGTGGTACTTGCG-3′	U69632
*TaNCY1*	F:5′-GAAATCCCTACCAAGCAATC-3′ R:5′-AATCCTCGGCAACATACTT-3′	JX398977
*TaNCY3*	R:5′-GACAAGGTGTTCTCGCGTATTA-3′ P:5′-GTGCCTCCTGAAAGGATATCTG-3′	JX398977
*TaDOS*	F:5′-TTCCGCATGTACGACTTC-3′ R:5′-GGAGTAGTGGTACTTGCG-3′	EF555121
*TaAKaGall*	F:5′-TGAGGCCTTCAACAGCATAG-3′ R:5′-CCGGCAGATGCAAGATATGA-3′	D86327
*Action*	F:5′-CAACCATAAACGATGCCGA-3′ R:5′-AGCCTTGCGACCATACTCC-3′	

### 2.9 Statistical analysis

The data of photosynthesis physiological and winter wheat growth yield were processed and statistically analyzed through Microsoft Excel 2010 and Sigma plot 14.0 software to process data and draw graphs, and DPS7.5 was used for statistical analysis. A two-way ANOVA was used to study the main influence and interaction of variable fluorescence types on yield. When there was a significant interaction effect between photosynthesis physiological, SPAD, and yield, the least significant difference (LSD) method was used for variance analysis and independent T test, and the significance level was set to α = 0.05. Differences were considered statistically significant when *p* ≤ 0.05.

## 3 Results

### 3.1 Photosynthetic characteristics

Net photosynthesis *P*_N_ reached the maximum value at the days after anthesis, and began to decline after the anthesis stage Figure [(1A)]. The maximum *P*_N_ value of 20–30 days was 19.09, which was increased by 16.41 % compared to of nitrogen rate respectively. The appropriate demographic structure was useful for accumulating net photosynthetic rates, and N240 significantly enhanced the *P*_N_ increase in winter wheat flag leaves there was significant difference between them in N0, and N180 a significant difference between the treatments in the after anthesis.

Effects of different N rate comparison of stomatal conductance *g*s Figure [(1B)]. The 20–30 days, N240 was the highest, reaching 0.45 followed by 20–30 days, N300, and 10–20 days N240. However, there was a significant difference between N300. N240 had a very significant effects on *g*_s_ at each growth stage, but they only N240 had a very significant interaction at the days after anthesis stage.

The transpiration rate (*Tr*) of each treatment overall trend of first increasing, and then decreasing Figure [(1C)]. The *Tr* value of N180 and N240 at the days after anthesis was greater than that of N0 and N300, and there was no significant difference between N0, and N300. The effects of N240 on *Tr* was the largest at the days after anthesis, so optimizing the combination of nitrogen application rate could enhance the leaf function of wheat at the late stage.

**Fig. 1:**
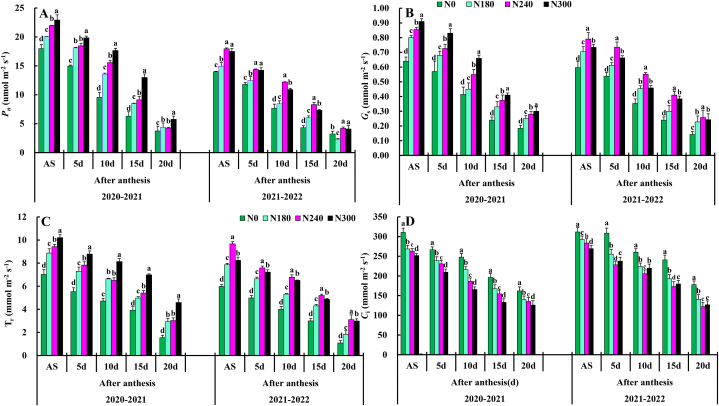
Effects of nitrogen application on anthesis stage (AS), net photosynthetic rate (*P*n), stomatal conductance (*g*_s_), transpiration rate (Tr), and Ci: intercellular CO_2_ concentration.

Effects of different nitrogen applications Figure [(1D)] the influence of the C_i_ winter wheat flag leaves. The C_i_ value of 10–20 days, N180 treatment at the days after anthesis, and N0 treatment might be due to the high nitrogen level (see Fig. 1).

### 3.2 Activitie enzymes

**Fig. 2:**
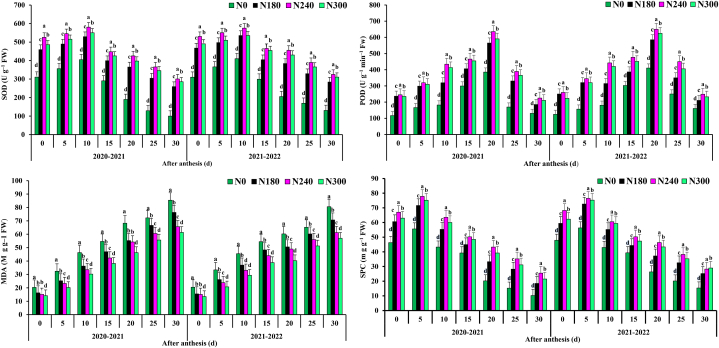
Effects of nitrogen application on fluorescence characteristic Superoxide dismutase activities (SOD), Peroxidase isozyme (POD), Malondialdehyde concentration (MDA), soluble protein concentration (SPC) in wheat flag leaves after anthesis.

At 0–15 days after anthesis, the activities of superoxide dismutase activities (SOD) and peroxidase (POD) and soluble protein in flag leaves of N0, N180 had no significant differences with those of N240 (Fig. 2). At 20–30 days after anthesis, the activities of superoxide dismutase (SOD) and peroxidase (POD) and soluble protein in flag leaves of N240 were significantly higher than those of N300, respectively. At 0–10 days after anthesis in flag leaves of N0, N180 wheat showed no significant difference, but at 15–30 days after anthesis in N240 was significant. N240 the superoxide dismutase (SOD) activity, peroxidase (POD) activity table, soluble protein in flag leaves of wheat increased first and then decreased. In the two wheat growing seasons, malondialdehyde (MDA) in flag leaf of wheat after anthesis increased and then decreased with the progress of grouting. At 0–15 days after anthesis, there was no significant difference in malondialdehyde (MDA) in flag leaves of N0 wheat. At 20–30 days after anthesis, (POD) activity of N240 was significantly lower than that of N300. The malondialdehyde (MDA) of wheat flag leaves decreased with the increase of nitrogen application rate. The activities of superoxide dismutase (SOD) and peroxidase (POD), soluble protein in the kernel flag leaf of wheat after anthesis first increased and then decreased with the progress of grout.

### 3.3 Root volume density

**Fig. 3:**
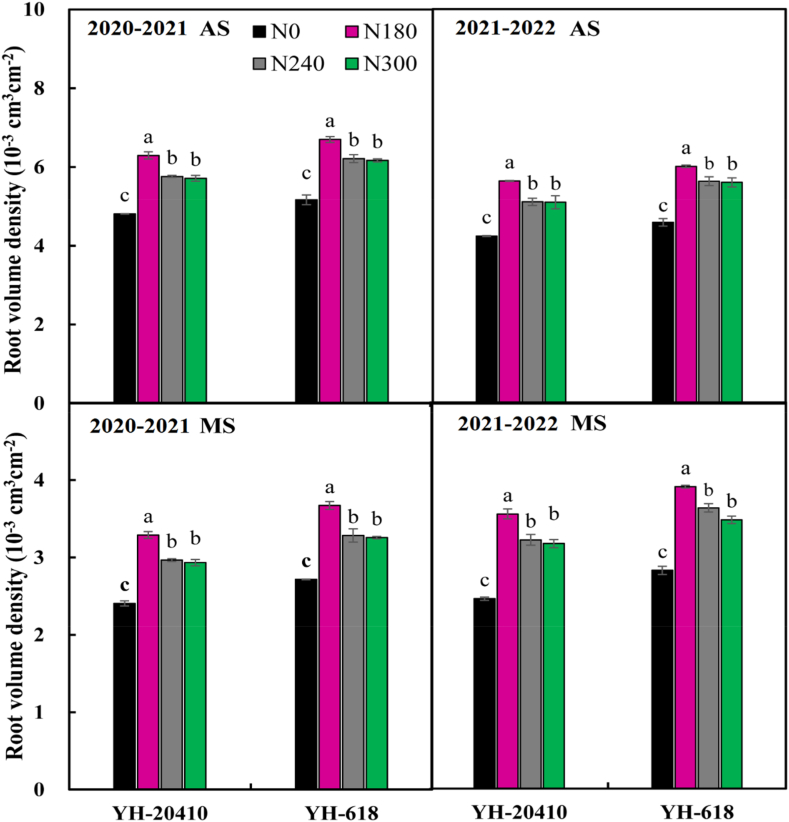
Effects of nitrogen rate on root volume density at anthesis (AS) and maturity (MS) (Upper-case letters and lower-case letters indicate comparisons between sowing methods and N rates between sowing methods. letter means significant at 0.05 probability levels. bars represent the standard error).

The YH-20410 significantly increased the root volume density soil layer at maturity stages 7.4 % 11.5 % (Fig. 3). The increase of N300 the root weight density in 0–60 cm soil layer increased firstly and then decreased significantly at anthesis and maturity stages, and the highest N180.

The nitrogen application rate had extremely significant effects on root activity at flowering stage, and the interaction between nitrogen application rates had extremely significant effects on root activity at flowering stage (Table 4). YH-20410 compare to the root activity of 0–20 cm, 20–40 cm and 40–60 cm soil layers at flowering stage was increased by 6.7–11.2 %, and 8.4 %–12.5 %, respectively. The increase of N300 the root activity in 0–60 cm soil layer increased firstly then decreased, and the highest nitrogen application rate was N240.

**Table 4:** Effects of nitrogen rate on triphenyl tetrazolium chloride (TTC) reduction activities in 0–60 cm soil layers at anthesis (μg g^−1^ FW h^−1^).

Year	Variety	N rate (kg ha^−1^)	Anthesis stage (AS)		
			0–20	20–40	40–60
2020–2021	YH-618	0	60.7c	53.3c	46.2c
		180	85.7a	75.6a	66.3a
		240	86.9a	76.6a	68.3a
		300	79.7b	68.3b	59.2b
		**Mean**	**78.3B**	**68.5B**	**60.0B**
	YH-20410	0	70.2c	65.4c	55.2c
		180	90.3a	80.1a	75.2a
		240	89.4a	79.7a	70.4a
		300	84.7b	70.6b	69.3b
		**Mean**	**83.7A**	**74.0A**	**67.5A**
2021–2022	YH-618	0	55.3c	40.3c	41.8c
		180	72.7a	62.3a	53.1a
		240	70.9a	61.0a	53.5a
		300	63.7b	54.8b	50.7b
		**Mean**	**65.7B**	**54.6B**	**49.8B**
	YH-20410	0	57.2c	53.6c	44.9c
		180	77.3a	66.5a	58.5a
		240	77.1a	67.1a	58.3a
		300	68.7b	60.1b	54.3b
		**Mean**	**70.1A**	**61.8A**	**54.0A**
ANOVA					
Year (Y)			**	**	**
Variety (V)			**	**	**
Nitrogen (N)			**	**	**
Y x V			ns	ns	ns
Y x N			ns	ns	ns
V x N			**	**	**
Y x V x N			**	**	**

### 3.4 Expression of aging genes in postanthesis wheat

**Fig. 4:**
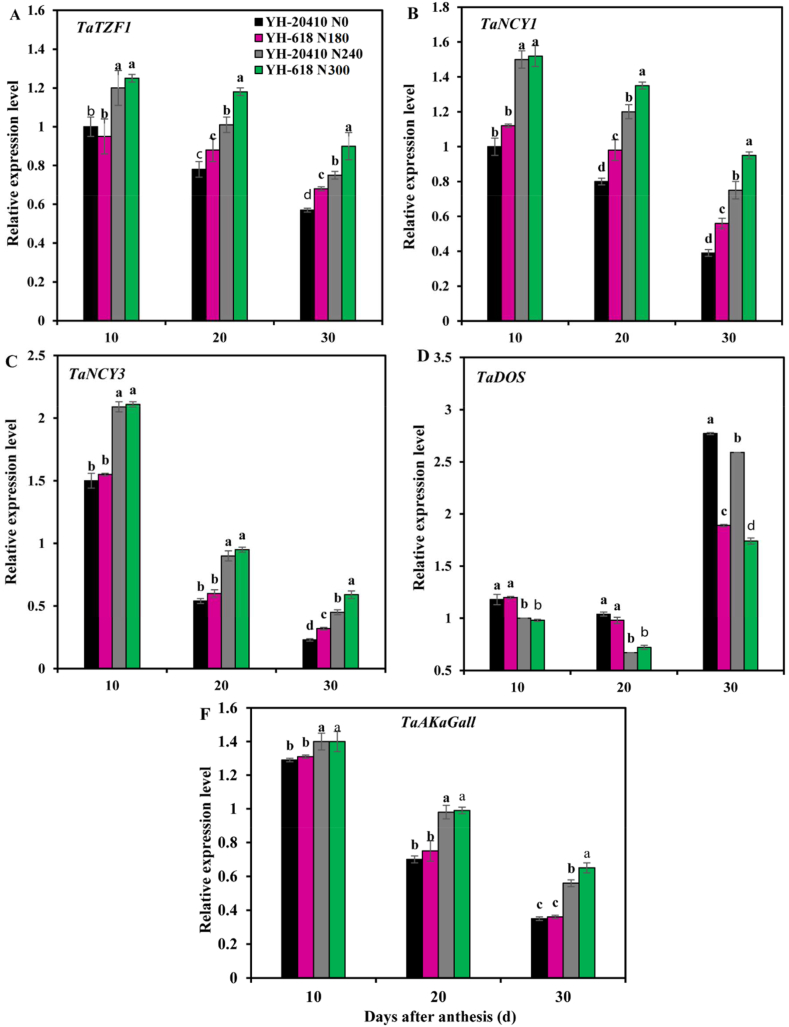
Effects of nitrogen rate on relative expression of genes leaf senescence at different stages after anthesis.

The expressions of *TaTZF1, TaNCY1, TaNCY3* and *TaAKaGall* genes in flag leaves of wheat with the variety were down-regulated of while *TaDOS* was up-regulated (Fig. 4). At 10 days after anthesis stage, there was no significant difference in the expression of *TaTZF1, TaNCY1, TaNCY3, TaDOS* and *TaAKaGall* genes in flag leaves of wheat between nitrogen treatments while the expression of these genes in flag leaves of wheat treated with N300 was significantly up-regulated compared to nitrogen treatment except *TaDOS*. At 20 days after anthesis, the expression of *TaTZF1*, and *TaNCY1* genes was significantly up-regulated in YH-618 compared to YH-20410 at 240 kg ha^−1^ but there was no significant difference in the expression of *TaNCY3, TaDOS* and *TaAKaGall* genes in flag leaves between variety and nitrogen treatments. The expression of these genes in nitrogen treatment was significantly up-regulated compared to no nitrogen treatment (except *TaDOS*). At 30 days after anthesis, the expression of *TaTZF1, TaNCY*, *TaNCY3* and *TaAKaGall* genes in flag leaves of YH-20410 wheat was N300 significantly up-regulated compared with that of YH-618, and the above genes were significantly up-regulated under nitrogen treatment compared with that without nitrogen treatment. The *TaDOS* gene expression in flag leaves of YH-20410 wheat was significantly down-regulated compared to YH-618 and the tados gene expression in flag leaves of wheat treated with nitrogen was significantly down-regulated compared to those treated without nitrogen.

### 3.5 Dynamic changes of fluorescence characteristics and flag leaves after anthesis

**Fig. 5:**
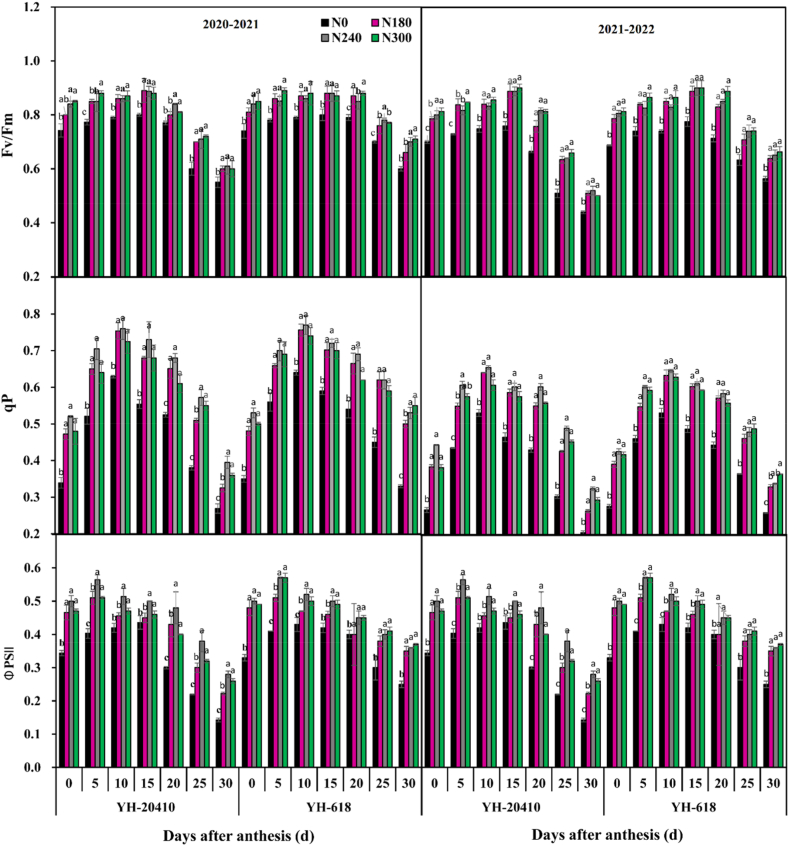
Effects of nitrogen rate on fluorescence characteristic in wheat flag leaves after anthesis. F_v_/F_m_, Maximum photochemical efficiency of q^p^ and non-photo-chemical quenching of ^Φ^_PSII_ reached the maximum value flag leaf in winter wheat.

The maximum potential photochemical efficiency of flag leaves of wheat after anthesis first increased and then decreased with grouting (Fig. 5). At 0–15 days after anthesis, the maximum potential photochemical efficiency of flag leaves in YH-618 compared to YH-20410 had no significant difference but at 20–30 days after anthesis, the maximum potential photochemical efficiency of flag leaves in YH-20410 was significantly higher than that in YH-618. The maximum potential photochemical efficiency of flag leaves increased with the increase of N300. The photochemical quenching coefficient and actual photochemical efficiency of flag leaves of wheat after anthesis first increased and then decreased with the progress of grout. At 0–20 days after anthesis, the photochemical quenching coefficient and actual photochemical efficiency of flag leaves in YH-618 and YH-20410 had no significant difference, but at 25–30 days after anthesis the photochemical quenching coefficient and actual photochemical efficiency of flag leaves in YH-20410 were significantly higher than those in YH-618. Increase of nitrogen application rate N300 the photochemical quenching coefficient efficiency and actual photochemical efficiency of flag leaves of YH-618 increased first and then decreased. The photochemical quenching coefficient efficiency, and the actual photochemical efficiency of flag leaves in YH-20410 showed an increasing trend.

**Fig. 6:**
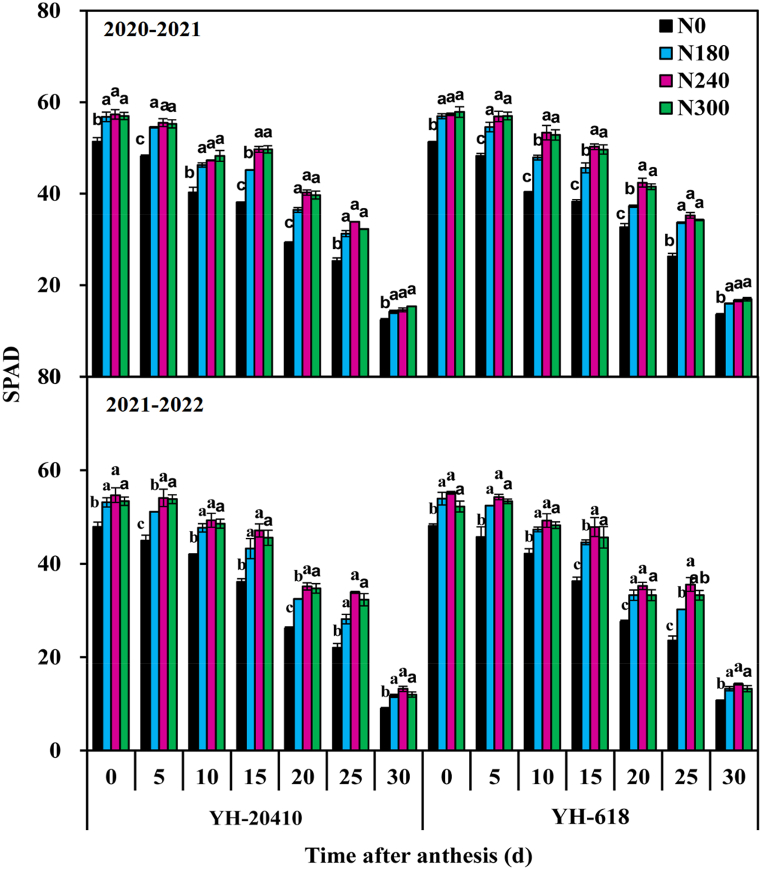
Effects of nitrogen rate on SPAD in wheat flag leaves after anthesis. Lower-case letters indicate comparisons between N rates means significant at 0.05 probability levels. Bars represent the standard error).

The relative content of chlorophyll in post-anthesis wheat decreased with the filling process (Fig. 6). At 0–15 days after anthesis there was no significant difference in the relative content of chlorophyl in flag leaves of YH-618 compared to YH-20410, but at 20–30 days after anthesis the relative content of chlorophyl in flag leaves of YH-20410 was significantly higher than that of YH-618. The increase of N300 the relative content of wheat chlorophyll in flag leaves showed an increasing trend.

### 3.6 Dry matter accumulation and yield composition

The nitrogen had extremely significant effects on dry weight at maturity, dry weight at anthesis dry matter accumulation after anthesis significant effects on dry weight at maturity dry weight at anthesis and dry matter accumulation after anthesis (Table 5). The increase of N240 dry weight at mature stage, and anthesis stage dry matter accumulation after anthesis were significantly increased. With the continuous increase of N240, N300 the dry weight at maturity dry weight at anthesis, and dry matter accumulation after anthesis increasing trend in YH-618, and YH-20410, while the dry weight at maturity, dry weight at anthesis, and dry matter accumulation after anthesis decreasing trend in YH-618. The concluded that YH-20410 can increase dry matter accumulation, and thus promote the increase of yield.

**Table 5:** Effects of nitrogen rate on dry matter accumulation.

Year	Variety	N rate (kg ha^−1^)	TDW (kg ha^−1^)	HI (%)	TDW_as_ (kg ha^−1^)	TDW_post_ (kg ha^−1^)
2020–2021	YH-618	0	9420.3c	47.0a	6417.0c	3003.3c
		180	16520.6b	42.0b	11052.8b	5467.8b
		240	17760.4a	41.5b	11838.2a	5922.1a
		300	16714.6b	41.0b	11216.7b	5497.9b
		**Mean**	**14921.1B**	**42.9A**	**10131.2B**	**4972.8B**
	YH-20410	0	10619.0d	46.0a	6829.8d	3789.2c
		180	18591.7c	42.6b	11872.6c	6719.1b
		240	19628.8b	42.1b	12400.9b	7227.9a
		300	20178.1a	41.9b	12927.9a	7250.2a
		**Mean**	**17254.4A**	**43.2A**	**11007.8A**	**6246.6A**
2021–2022	YH-618	0	7239.9c	51.0a	4625.0c	2614.9c
		180	13419.1b	46.2b	8885.5b	4533.6b
		240	14155.7a	45.7b	9385.4a	4770.3a
		300	13134.0b	45.2b	8605.2b	4528.8b
		**Mean**	**11987.2B**	**47.0A**	**7875.3B**	**4111.9B**
	YH-20410	0	8657.3d	50.0a	5498.4d	3158.9c
		180	14100.0c	45.9b	8956.2c	5143.8b
		240	15143.2b	45.6b	9605.6b	5537.6a
		300	15697.7a	45.6b	10098.9a	5598.8a
		**Mean**	**13399.5A**	**46.8A**	**8539.8A**	**4859.8A**
ANOVA						
Year (Y)			**	**	**	**
Variety (V)			**	ns	**	*
Nitrogen (N)			**	**	**	**
Y x V			**	*	**	**
Y x N			ns	ns	ns	ns
V x N			ns	ns	ns	ns
Y x V x N			**	**	**	**

Note: TDW_as_, Total dry weight anthesis stage; TDW, Total dry weight; HI, Harvest Index.

The number of spike, grain number per ear, and yield were significantly affected the interaction between nitrogen had significantly affected the number of spike, grain number per ear, 1000-grain weight and yield (Table 6). YH-618 spike number, and yield were significantly increased by 18.8%–19.7 % and 13.6%–16.3 % under YH-20410. The increase of N240 spike number, yield and grain number per spike were increased, while 1000-grain weight was decreased. The continuous increase of N240, N300 spike number increased while 1000-grain weight and grain number per spike decreased while the yield of YH-20410 an increasing while of YH-618. At the same time the yield of N180 was not significantly different from that of N240.

**Table 6:** Effects nitrogen rate on grain yield and yield components.

Year	Variety	N rate (kg ha^−1^)	Ear number (10^4^ ha^−1^)	Grain number per ear	1000 grain weight (g)	Yield (kg ha^−1^)
2020–2021	YH-618	0	377.2d	22.2c	43.2a	3154.6d
		180	480.0c	26.5a	41.9b	4972.2b
		240	525.0b	26.3a	40.9b	5440.4a
		300	557.3a	24.1b	38.3c	4821.6b
	YH-20410	0	434.0d	21.1c	43.1a	3671.8c
		180	577.5c	25.0a	41.6b	4000.8b
		240	613.0b	25.8a	41.1b	5593.2a
		300	680.1a	23.5b	40.8c	5778.5a
2021–2022	YH-618	0	290.3d	21.9c	42.5a	3450.1c
		180	438.0c	26.2a	40.2b	4857.0b
		240	462.3b	26.9a	40.1b	5340.7a
		300	481.3a	23.3b	37.1c	4721.4b
	YH-20410	0	358.3d	20.7c	42.1a	3868.9c
		180	500.5c	25.4a	39.9b	4450.8b
		240	540.5b	25.1a	39.8b	5095.4a
		300	600.3a	23.3b	38.1c	5225.6a
ANOVA						
Year (Y)			**	**	**	**
Variety (V)			*	**	ns	**
Nitrogen (N)			**	**	**	**
Y x V			ns	ns	ns	ns
Y x N			ns	ns	ns	ns
V x N			**	**	*	**
Y x V x N			**	**	**	**

### 3.7 Relationship between leaf senescence genes and yield, dry matter nitrogen accumulation

The expression levels of *TaNCY3* and *TaAKaGall* genes at 10 days after anthesis were significantly correlated with yield, maturity nitrogen and postanthesis nitrogen accumulation (Table 7). The expression levels of *TaTZF1, TaNCY1,* and *TaNCY3* genes at 20 and 30 days after anthesis were significantly correlated with yield, dry weight at maturity and dry weight accumulation after anthesis. At 30 days after anthesis, *TaAKaGall* gene expression was significantly correlated with yield, dry weight accumulation after anthesis, dry weight at maturity, nitrogen accumulation at maturity and nitrogen accumulation after anthesis. In conclusion, the expression of senescence genes in the middle, and late stages was beneficial to the accumulation of dry matter and nitrogen, and promoted the increase of yield.

**Table 7:** Correlation analysis of genes leaf senescence with yield and dry matter accumulation in the growth period.

Index		Yield	Total dry weight	Total dry weight anthesis	TN_ms_	N_post_
*TaTZF1*	10 AS10	0.4512	0.4211	04122	0.4122	0.4515
	20 AS20	0.9012**	0.9451**	0.9122**	0.8925**	0.5515*
	30 A30	0.8712**	0.8515**	0.9022**	0.9022**	0.8415**
*TaNCY1*	10 AS10	0.4212	0.4912	0.4822	0.4302	0.4615
	20 AS20	0.8212**	0.8123**	0.8512**	0.9125**	0.8015**
	30 A30	0.9162**	0.9003**	0.9111**	0.9100**	0.8614**
*TaNCY3*	10 AS10	0.8312**	0.8422**	0.8511**	0.9021**	0.8115**
	20 AS20	0.8112**	0.8222**	0.8222**	0.8622**	0.8312**
	30 A30	0.9215**	0.8512**	0.5301*	0.7112**	0.7412**
*TaDOS*	10 AS10	0.4212	0.4011	0.4312	0.4542	0.4625
	20 AS20	0.4622	0.3811	0.4002	0.3981	0.4024
	30 A30	−0.5212*	−0.4212	−0.3212	0.4281	0.4356
*TaAKaGall*	10 AS10	0.9212**	0.5311*	0.8312**	0.8562**	0.9625**
	20 AS20	0.4822	0.4819	0.4609	0.4981	0.4624
	30 A30	0.7622**	0.8811**	0.8202**	0.9601**	0.8224**

Notes: TN_as_, Total nitrogen at maturity stage * and ** denote significant correlation at 5 % and 1 % probability levels, respectively.

## 4 Discussion

### 4.1 Effects of nitrogen application on root physiological characteristics

Wheat root system is the main organ that affects the absorption and utilization of soil nutrients and the growth and development of wheat [24]. The YH-20410 could significantly increase the root weight density, root length density. Root surface area density in 0–60 cm soil layer at flowering and maturity stage compared with plain YH-618, which was conducive to the growth and development of roots [25]. The transpiration rate and net photosynthetic rate N150 of decreased significantly grain weight yield [26]. Photosynthesize single photon avalanche diode SPAD characteristics, and photosynthesis characteristics were higher than those of the N120, which were consistent with the results of previous studies. Thus, we used four nitrogen application concentrations set as N120, and N150 respectively to analyze the photosynthetic characteristics, and yield-related traits in winter wheat. Therefore, the single photon avalanche diode SPAD with N120 kg ha^−1^ would be a valuable management practice to improve wheat yield. The premise of further reducing the amount of nitrogen application at the jointing stage could improve grain quality, and increase nitrogen use efficiency [27]. The nitrogen uptake ratio of different special types of wheat was different in each growth period, and the nitrogen uptake, and nitrogen uptake ratio of wheat were the largest from jointing to flowering [28]. The retransfer of wheat nitrogen from vegetative organs to grains is restricted by environmental factors, and Photosynthetic differences. In the process of nitrogen application different varieties will not only affect the effects of nitrogen application, but also cause the difference in nitrogen absorption, accumulation, and transport as well as dry matter accumulation, distribution, and yield formation of crops. Therefore, only the combination of appropriate nitrogen regulation rational planting measures to coordinate the contradiction between crop nutrient absorption, and nitrogen use efficiency can improve the nitrogen use efficiency of wheat, and achieve high yield [29]. Nitrogen is an important component of photon avalanche diode SPAD various dry gluten and approximately 75 % of the nitrogen in plant leaves occurs in SPAD most of which are used to construct photosynthetic apparatus [29]. The ratio of variable fluorescence to maximum fluorescence (F_V_/F_m_) reflectsthe maximum photosynthetic quantum yield of ΦPSII. F_V_/F_m_ is not affected by species, andthis parameter changes little under non-stress conditions. Under stress F_V_/F_m_ values decreased signifi-cantly, and the potential photosynthetic activity of ΦPSIIwas inhibited. Water stress inhibits photosynthetic electrontransfer and photosynthetic membrane energy quantization. It has been reported that the long term response to CO_2_ concentrations is mainly due to the limitation of nitrogen supply [8]. Many experiments have also detected a decrease in plant nitrogen concentration under CO_2_ enrichment conditions. The leaf is an important component of the later growth stage contributing about 30 % to the photosynthesis of the population wheat [29]. YH-20410 significantly increased root length density, and root volume density in 0–60 cm soil layers at flowering and maturity stages, increased root superoxide dismutase activity and root reduction intensity in 0–60 cm soil layers at flowering stage, and decreased root malondialdehyde content in 0–60 cm soil layers. That fertilization had a significant effect on the regulation of wheat root growth, and nitrogen application rate was N150 kg ha^−1^, could significantly improve the dry weight of underground roots. Nitrogen application rate can promote the formation of underpinning or lateral roots of wheat roots [30,31]. The absorption and utilization of soil water and nitrogen by roots. When the nutrient supply of soil is sufficient, the nutrients absorbed by wheat roots are preferentially supplied to overshoot plants, so the growth of overshoot plants is faster than that of roots in underground parts, and the root-shoot ratio decreases [32]. Crops allocate more carbohydrates to their roots to increase crop root surface area [33,34]. The low N treatment N180 kg ha^−1^ high N treatment (N240 kg ha^−1^ and N300 kg ha^−1^) reduced root weight density, root length density and root volume density. The decrease of root weight density, root length density and root volume density under high nitrogen conditions is mainly related to the distribution of carbohydrate in the aboveground and underground parts of crops. The aboveground and underground parts of plants have feedback regulation between them [35]. In this experiment, there was no significant difference between the relative content of chlorophyll in flag leaves of YH-20410 wheat at 0–15 days after anthesis, but the relative content of chlorophyll in N240 kg ha^−1^ days after anthesis. YH-20410 can delay the degradation of leaf chlorophyll properly. In the later stages of wheat growth, the carbohydrate production of wheat flag leaves increases by about 60 %, and the late growth products account for about 70–80 % of crop yield [36]. Therefore, the photosynthetic capacity of post-flowering flag leaves of wheat can represent the photosynthetic characteristics of the overall canopy of wheat [37]. In this study was N300 kg ha^−1^ significantly higher in YH-20410 than in YH-618 F_v_/F_m_ reflects the efficiency of light energy conversion in the ФPSII reaction center, and its size and variation characteristics can be used to judge the resistance performance of plants to external environmental factors [25,38]. In this experiment, different nitrogen rate had significant effects on the chlorophyll fluorescence parameters of flag leaves in the late growth stage of wheat. The maximum potential photochemical efficiency (F_v_/F_m_) and actual photochemical efficiency qP of ФPSII under YH-20410 were significantly higher than those under conventional treatment, which was conducive to the photoreaction of flag leaves ФPSII the production of more photosynthates, and the increase of yield. Correlation analysis also showed that yield was significantly correlated with net photosynthetic rate, transpiration rate, maximum potential photochemical efficiency and photochemical quenching coefficient of flag leaves at 10–30 days after anthesis. In this experiment, high nitrogen fertilizer was not conducive to the improvement of the above photosynthesis and fluorescence indices, and YH-20410 with N240 kg ha^−1^ was better.

### 4.2 Effects of nitrogen application on gene expression

The yield of wheat per unit area will further increase with the increase of yield composition. However, in actual field production, due to the changes of cultivation external growth environment. The three factors of wheat yield are difficult to achieve synergistic growth [12]. Deep soil water utilization of winter wheat is an important means to reduce irrigation and improve grain yield [39]. Nitrogen is one of the most important limiting factors for crop growth development and improving crop utilization supply of nitrogen for crop yield is crucial for sustainable development of agriculture. Studies have shown that nitrogen absorption efficiency is the most important factor determining nitrogen absorption, especially under low nitrogen application conditions [19]. This is mainly related to the characteristics of wheat root system, and depends on the structure and function of wheat root system. In agricultural soils nitrogen absorption is regulated by wheat demand for dry matter accumulation [25,36]. Plant senescence is mainly affected by complex gene regulatory pathways, and gene expression products can directly or indirectly act on a specific part of plant leaves, thus regulating senescence and death of plant leaves [17]. Relevant studies have shown that *TaTZF1* gene plays a significant role in the process of crop coping with adverse environment and can delay leaf senescence [40]. *TaDOS* gene is a gene that accelerates leaf aging [41]. *TaAKaGall* gene is a gene that delays plant aging [42]. The results of the field test showed that the expression of *TaTZF1, TaNCY1*, *TaNCY3* and *TaAKaGall* genes in the flag leaves of YH-20410 wheat was significantly up-regulated compared with that of YH-618 at the late stage of filling 30 days after anthesis, and the above genes were significantly up-regulated under nitrogen treatment compared to that without nitrogen application. The *TaDOS* gene expression in flag leaves of YH-20410 wheat was significantly down-regulated compared to YH-20410, and the Tados gene expression in flag leaves of wheat treated with nitrogen was significantly down-regulated compared with those treated without nitrogen. Correlation analysis also showed that the expression levels of *TaTZF1, TaNCY1* and *TaNCY3* genes at 20 and 30 days after anthesis were significantly correlated with yield dry weight at maturity and dry weight accumulation after anthesis. It is concluded that YH-20410 was beneficial to delay the senescence of wheat leaves in the later stage is beneficial to the accumulation of dry matter and nitrogen, and promotes the increase of yield.

### 4.3 Effects of nitrogen application on yield quality

Wheat Protein grains accounts for about 10 %, 15 % of the total grain amount, which is an important nutrient in wheat and also an important factor determining the processing quality of wheat grains [43]. Most of the nitrogen required for crop growth and development is obtained by plants directly absorbing soil nitrogen through their roots. The temporal and spatial distribution of crop roots in soil will directly affect the utilization of soil water and nutrients by wheat [44]. Wheat roots have strong plasticity and self-regulation ability and are greatly influenced by the regulation effect of cultivation measures [45]. There was changes in external environmental conditions and cultivation measures not only affect the protein content of wheat grains, but also affect the protein composition of wheat grains [46]. Studies have shown that appropriate increase of nitrogen fertilizer can significantly increase wheat grain protein content [15,47]. The increase of N240, N300 the grain protein content of YH-20410 sowing at maturity stage increased significantly, while the grain protein content. The protein contents were higher in N240 treatment. Starch is the most important component in wheat grain, which is mainly composed of amylose and amylopectin. Starch accounts for 60%–70 % of dry grain weight of mature wheat [47,48]. There are few studies on the effect of nitrogen fertilizer on starch content in wheat grains. Nitrogen fertilizer regulates the synthesis of grain starch by regulating wheat photosynthetic carbon assimilation, accumulation, transport and distribution of carbohydrates, and further affects the quality of wheat grain starch [47,49]. The nitrogen application could improve the transport of dry matter accumulated in vegetative organs to grains. The same time improve the activity of key enzymes for starch synthesis in grains, thus promoting starch synthesis and accumulation in wheat grains [50]. However, high nitrogen levels are not conducive to the redistribution of dry matter in vegetative organs and reduce the accumulation of starch [51]. In this experiment, amylose, amylopectin, total starch and straight/branch ratio all decreased with the increase of nitrogen application rate N0, N300. F_v_/F_m_ reflects the efficiency of light energy conversion in the ФPSII reaction center size and variation characteristics can usually be used to judge the resistance of plants to external environmental factors [52]. Therefore, the complementary effect between yield components has always been a major obstacle to the improvement of wheat yield [53]. Previous studies have found that the higher the crop yield, the more obvious changes in the relationship between spike number, grain number per spike and 1000-grain weight. Spike number is the leading factor for coordinating yield composition, and increasing spike number will lead to higher yield [23]. There are many previous studies on the effects of nitrogen application on wheat yield and its composition. It was found that the number of ears and grains per spike per increased with the increase of nitrogen application rate, while the 1000-grain weight of wheat decreased with the increase of nitrogen application rate [54]. Wheat yield was the highest N300 kg ha^−1^, and the effects of increasing nitrogen application rate was not significant [55]. The range of N90 grain number per spike and 1000-grain weight of wheat increased with the increase of N application rate. When nitrogen application rate exceeded N240 grain number per spike of wheat did not increase significantly. When nitrogen application rate exceeded N180, 1000-grain weight of wheat did not increase significantly [56]. Found that excessive application of nitrogen fertilizer would significantly reduce the number of spike and 1000-grain weight of wheat increase the number of grains per spike. However, excessive nitrogen application, although it can significantly improve the tillering of wheat, can increase the number of ears per unit area, easy to cause late ripening and lodging of wheat, and affect the number of grains per ear and grain weight, resulting in a decrease in wheat yield [2]. In this experiment, high nitrogen fertilizer was not conducive to the improvement of the above photosynthetic and fluorescence indexes, and N240 with variety YH-20410 was better. The increase of N0, N300, all flour quality indexes showed an increasing trend. High yield, and high quality can be achieved by variety YH-20410.

## 5 Conclusion

This study suggests that values of variety YH-20410 mainly increased wheat yield by increasing the number of ears. The increase of spike number was mainly through the increase of tiller number. The yield increase of N240 homogenized wheat was mainly dependent on higher dry matter accumulation rather than harvest index. The increase of dry matter accumulation mainly depended on wheat root growth and development. Variety YH-20410 combined to proper nitrogen fertilizer treatment can improve the antioxidant capacity of wheat roots, delay the senescence of roots, and promote the absorption and utilization of soil water and fertilizer by roots. YH-20410 compared to YH-618 could improve the canopy light energy blocking amount, further the photosynthetic and fluorescence characteristics of post-flowering flag wheat leaves, improve the enzyme activity of antioxidant system, cause significantly up-regulated expression of anti-aging *TaTZF1, TaNCY1, TaNCY3* and *TaAKaGall* gene in post-flowering wheat, and delay the post-flowering leaf senescence. Under the conditions of this experiment N180 combined with nitrogen can ensure high yield, high quality and high resource utilization efficiency under the condition of Variety YH-20410. The increase of dry matter accumulation mainly depended on the growth and development of wheat root system.

## Funding

This project was approved by the Scientific and Technological Innovation Programs of Higher Education Institutions in Shanxi (2019L0893) for financial support of this study.

## Data availability statement

Data will be made available on request.

## CRediT authorship contribution statement

**Bo Qu:** Investigation, Funding acquisition, Formal analysis. **Fujie Feng:** Validation, Supervision, Software, Resources. **Jun Di:** Investigation, Funding acquisition, Formal analysis. **Hafeez Noor:** Writing – review & editing, Writing – original draft, Project administration, Funding acquisition, Data curation.

## Declaration of competing interest

The authors declare that they have no known competing financial interests or personal relationships that could have appeared to influence the work reported in this paper.
